# Supplementary material for: Machine vision model for detection of foreign substances at the bottom of empty large volume parenteral
Source: PLoS One. 2024 Apr 26;19(4):e0298108. doi: 10.1371/journal.pone.0298108 (PMC11051628; doi:10.1371/journal.pone.0298108)
Supplement: S1 File — (DOCX) [file pone.0298108.s001.docx]

Principle of image preprocessing algorithm.

Feature enhancement can increase the contrast information of the object to be recognized in the background, ensuring a clear distinction between foreign objects and the clean bottom area of the bottle. Its principle is shown in equation(1)：

$$I_{em}=\mathrm{round}((I-mean_{m\times m})*K_{em})+I$$

Formula (1) can further enhance the background and contrast of the image. The enhanced image can then be used for defect extraction and detection through an adaptive global threshold segmentation algorithm. Further analysis of blob features can be performed to label the defects, based on the principles presented in equations (2) and (3).
